# Probiotics and Commensal Gut Microbiota as the Effective Alternative Therapy for Multiple Sclerosis Patients Treatment

**DOI:** 10.3390/ijms232214478

**Published:** 2022-11-21

**Authors:** Angela Dziedzic, Joanna Saluk

**Affiliations:** Department of General Biochemistry, Faculty of Biology and Environmental Protection, University of Lodz, Pomorska 141/143, 90-236 Lodz, Poland

**Keywords:** probiotics, bacterial-derived metabolites, gut-brain axis, depression, multiple sclerosis, adjuvant therapy

## Abstract

The gut-brain axis (GBA) refers to the multifactorial interactions between the intestine microflora and the nervous, immune, and endocrine systems, connecting brain activity and gut functions. Alterations of the GBA have been revealed in people with multiple sclerosis (MS), suggesting a potential role in disease pathogenesis and making it a promising therapeutic target. Whilst research in this field is still in its infancy, a number of studies revealed that MS patients are more likely to exhibit modified microbiota, altered levels of short-chain fatty acids, and enhanced intestinal permeability. Both clinical and preclinical trials in patients with MS and animal models revealed that the administration of probiotic bacteria might improve cognitive, motor, and mental behaviors by modulation of GBA molecular pathways. According to the newest data, supplementation with probiotics may be associated with slower disability progression, reduced depressive symptoms, and improvements in general health in patients with MS. Herein, we give an overview of how probiotics supplementation may have a beneficial effect on the course of MS and its animal model. Hence, interference with the composition of the MS patient’s intestinal microbiota may, in the future, be a grip point for the development of diagnostic tools and personalized microbiota-based adjuvant therapy.

## 1. Introduction

The human gastrointestinal tract is one of the biggest connections between the host, environmental agents, and antigens in the human body. The number of microorganisms living in the human intestinal tract has been rated to exceed 10^14^ [1], which encompasses approximately 10-fold more bacterial cells than human cells [2]. Consolidated data from the Human Microbiome Project (HMP) and the METAgenomics of the Human Intestinal Tract (MetaHIT) consortium provided the most comprehensive view of the microbial stock, showing that as many as 2766 microbial species exist in the human body [3,4,5,6]. So far, 12 different enterotypes of bacteria have been categorized in humans headed by Actinobacteria, Bacteroidetes, Firmicutes, and Proteobacteria, of which more than 90% constitute the intestinal microbiome [7].

Probiotics are defined as live microorganisms that, ingested in adequate amounts (FAO/WHO 2002), can affect the composition of gut microbiota and bacterial-derived metabolite production, as well as provide beneficial effects on immune diseases [8]. The immunomodulatory properties of probiotics and their favorable role in autoimmune and inflammatory diseases were numerously reported [9,10,11]. The intestine microbiota offers plenty of advantages to the host, by a scope of physiological functions, including maintaining gut integrity, barrier function, proper absorption of nutrients (well-nutrition of the body), protecting against pathogens, cell-to-cell signaling, and immunity [12]. Moreover, the gut microbiota may affect many aspects of brain development and function, including microglia and astrocyte maturation and polarization, regulating neurotransmission, neurogenesis, and myelination [13]. What is more, many reports refer to the beneficial effect of probiotics on improving mental state and cognition [14].

Probiotics have been recognized as one of the key elements affecting brain functioning, having a decisive influence on the development of mental disorders; therefore, they have gained the name ‘psychobiotics,’ while the gut microflora has been recognized as the ‘second brain.’ The term psychobiotics was first used in 2013 by clinical psychiatrist Professor Ted Dinan and neurologist Professor John Cryan. It refers to all probiotic microorganisms consumed in adequate amounts that can affect the immune system and have favorable effects on mental health and neurological functions, such as mood, anxiety, attention, memory, and cognition [15]. The term was used further by other researchers to designate beneficial microorganisms and bacteria-derived metabolites that indirectly influence mental health conditions and can be successfully administered as assisted therapy in the prevention and treatment of both neurological and psychiatric diseases [16,17,18].

Available clinical, epidemiological, and immunological evidence implies that the intestine microbiota extensively and profoundly affects the gut-brain relationship, affecting mental health, mood regulation, and motor function [19]. Several studies on brain-related disorders elucidated that probiotics and their bacterial-derived metabolites could potentially moderate the gut-brain axis (GBA).

The GBA is the bidirectional interaction between the gut microbiome and the central nervous system (CNS), being a complicated network of relationships among the intestine nervous system, sympathetic and parasympathetic nervous systems, as well as endocrine and immune systems [20]. There are different pathways of communication between the gut and the CNS, which include the autonomic nervous system (enteric nervous system (ENS) and the vagus nerve), the neuroendocrine system (hypothalamic–pituitary–adrenal axis (HPA)), metabolic pathways, and the immune system [21]. Both the enteric and vagus nerves are involved in gut-brain interaction, and their function may be modulated by certain probiotic bacteria [22]. Signaling is from the intestines to the CNS and, conversely, either directly via the autonomic nervous system or indirectly through the metabolites and neurotransmitters [21]. GBA alterations may participate in the pathophysiology of several brain disorders, including multiple sclerosis (MS) [23].

There is a growing body of reports that show that bacterial metabolites play a critical role in the regulation of immunity and mental state. The gut microbiota is also an abundant source of crucial metabolites needed for GBA signaling, such as neuroactive tryptophan (TRP), gamma-aminobutyric acid (GABA), histamine, serotonin (5-HT), and acetylcholine, as well as microbial metabolites that affect the immune response, such as short-chain fatty acids (SCFAs) derived from the fermentation of dietary fiber; it also plays a key role in the production of the components of the local mucosal immune system, cytokines, and neurotransmitters. SCFAs, especially butyrate, acetate, and propionate, have anti-inflammatory and neuroprotective properties, maintain intestinal barrier integrity, act as hormonal regulators, affect vagal afferents (a GBA neural component that allows bottom-up passage of information from the viscera to the CNS) [16,19]. A decrease in SCFAs could result in impaired remyelination, increased neuroinflammation, elevated blood-brain barrier (BBB) and gut barrier permeability, oxidative stress, and exacerbation of depressive symptoms in the course of MS [24]. In vitro studies suggest that SCFAs may affect myelination and Tregs expansion in the gut, independently of microglia in the CNS [25].

It is well-known that the gut microbiota may have a considerable impact on CNS health and disease. However, most of the information about the impact of gut microbiota on CNS changes is derived from studies in animal models, where researchers can effectively control the environment of the study animals. Even though mechanisms underlying GBA communication are still vague, it is now believed that the gut microbiome can affect CNS development and function, including immune cell maturation, BBB formation, neurogenesis, and myelination [26]. Intestine microflora is the essential agent underpinning CNS signaling and is an active contributor to the homeostatic processes. At the same time, the CNS controls most physiological processes ongoing in the gastrointestinal tract [27].

**Figure 1:**
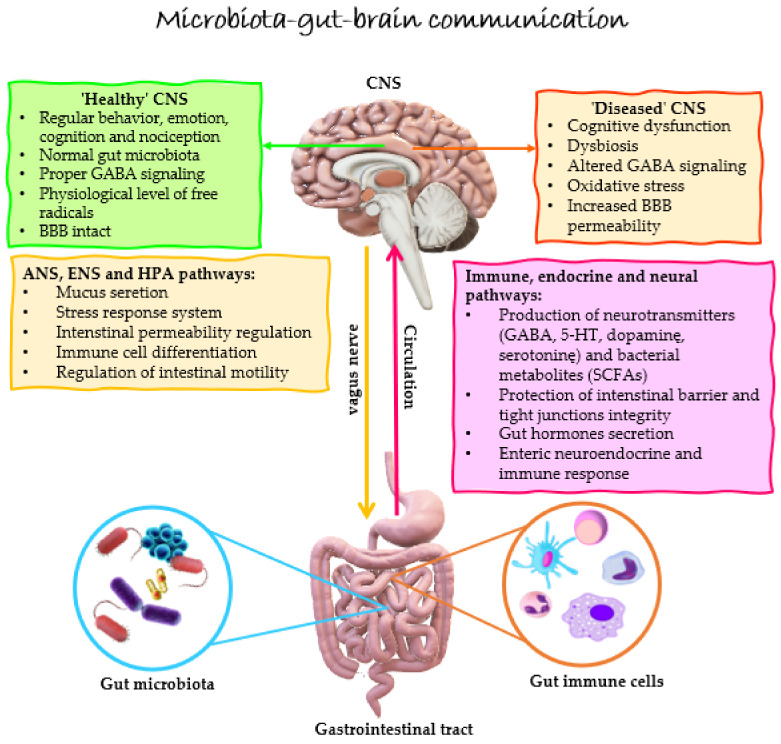
Schematic diagram showing the microbiota-gut-brain communication. Bidirectional interaction between the gut microbiota and the CNS is mediated by a few pathways of the GBA, including ANS, ENS, HPA, and the immune, neuroendocrine, as well as neural pathways. Within the intestines, the gut microbiota can produce neuroactive compounds, such as neurotransmitters (e.g., GABA, dopamine, and 5-HT) and microbial-derived metabolites (e.g., SCFAs). Altered gut microbiota (dysbiosis) may impair CNS activity, which is manifested by declined cognitive function, altered GABA signaling, enhanced free radicals level, and increased barriers permeability (BBB and gut barrier). Abbreviations: 5-HT—serotonin; ANS—autonomic nervous system; BBB—blood-brain barrier; CNS—central nervous system; ENS—enteric nervous system; GABA—gamma-aminobutyric acid; GBA—gut-brain axis; HPA—hypothalamic–pituitary–adrenal; SCFAs—short-chain fatty acids.

Several potential pathways indicate that probiotic bacteria may have a positive effect on the functioning of the CNS. First, probiotic bacteria may directly change CNS biochemistry by affecting brain-derived neurotrophic factor (BDNF) [28,29], GABA [30], 5-HT [31], and dopamine [32] levels, influencing the mind and behavior. Furthermore, it has been shown that bacterial-derived metabolites can regulate the maturation and activity of microglia, thereby affecting CNS functioning [33]. Another presumable pathway by which gut microbiota can influence the CNS is through variations in mature hippocampal neurogenesis [34]. However, a great deal of controversy exists regarding the exact molecular mechanism by which an altered gut microbiome could influence the development of inflammation in the CNS, demyelination, and axonal loss. Complex microbiota-gut-brain interactions are represented in Figure 1.

Given the essential participation of intestine bacteria in immune regulation via the GBA, it is believed that any variations in the gut microbiota are likely to influence brain-related diseases. Indeed, an increasing body of evidence from studies on animal models and patients indicates that the two-way interactions between the immune system and gut microbiota are crucial in the pathogenesis and progression of MS. Herein, we report an extensive overview of an influence of the different probiotic bacteria strains supplementation, both on MS patients and it’s animal model.

## 2. Epidemiology and Pathogenesis of MS

MS is an autoimmune disease characterized by chronic inflammation, demyelination, and neurodegeneration of the CNS [35]. Up to 2.8 million people worldwide suffer from MS (35.9 per 100,000 population), and its prevalence has raised in every world region annually [36]. MS affects young adults between 20 and 40 years old, but it can onset at any age. Furthermore, women are more susceptible to MS, at a commonly reported ratio of 3:1 over men or even higher [37]. In most relapsing-remitting (RRMS) patients (approximately 85%), after several years, develop into a progressive phase of MS, which is characterized by an irreversible disability and continuous disease progression without remission [38].

MS is an incurable and destructive disease with a variable clinical outcome and a high risk of disability [39]. The complicated disease processes result in brain and spinal cord atrophy, resulting in irreversible disablement and multi-organ dysfunction [40]. MS is characterized by cognitive impairment, dyskinesia, muscle spasticity, numbness, fatigue, depression, sexual dysfunction, anxiety, vision loss, dizziness, and gastrointestinal dysfunction [41]. Of relevance, there is a robust body of evidence showing that neuropsychiatric disorders, including major depressive disorders (MDD), are among the most common comorbidities (approximately 50%) in people with MS compared to the general population [42,43,44].

Currently, various disease-modifying therapies (DMTs) are available for the treatment of MS, including orals (fingolimod, teriflunomide, dimethyl fumarate), injectable (interferons, glatiramer acetate, mitoxantrone), and monoclonal antibodies (natalizumab, alemtuzumab, daclizumab, ocrelizumab). The initiation of DMT at an early stage of MS diagnosis may significantly ameliorate the prognosis for these patients and diminish the occurrence of neurological damage [45]. However, none of the DMTs used for the management of MS have been fully effective so far. On top of this, many harmful side effects associated with the use of DMTs have restricted the practice of combination therapy. Thus, there is a great need to use safe immunomodulatory factors as adjuvant therapy in MS management.

The pathological hallmarks of MS are the disruption of the BBB, oligodendrocyte loss, demyelination, microglial activation, astrocyte proliferation, and axonal degeneration [46]. Although the exact molecular mechanisms of MS development and progression are still ambiguous, it is assumed that MS pathogenesis is mediated via an autoimmune response where the T and B cells become key players.

The development of MS is considered to be an interaction of genetic predisposition, environmental factors, and aberrant immune response, but the precise etiology of the disease remains unknown [47]. Also, studies have suggested that oxidative stress may play an important role in the pathogenesis of MS [48] as one of the common features in the brain of MS patients is the imbalance between oxidants and antioxidants with increased concentrations of reactive oxygen species (ROS) in cerebrospinal fluid (CSF) of MS patients [49]. Furthermore, clinical studies have shown increased oxidative stress in the blood of MS patients, including dysregulated malondialdehyde (MDA) [50], superoxide dismutase (SOD) [51], and glutathione (GSH) [52] levels in the patients.

The impaired immune response in MS is probably triggered by a shift in the balance between pathogenic T helper (Th)1/Th17 cells and regulatory cells such as T regulatory (Treg) cells and B regulatory (Breg) cells. The immunopathology of MS is mainly characterized by increasing the number of pro-inflammatory cells, including CD4^+^ T cells with the Th1 or Th17 phenotypes, monocytes (CD14^+^/CD16^+^), macrophages, dendritic cells, and B cells, and a decrease in the level of Th2, CD3^+^/CD8^+^ T cytotoxic (Tcyt) cells and regulatory cells, such as Tregs (CD4^+^/CD25^+^/FoxP3^+^) and Bregs (CD19^+^/CD5^+^/Cd1d^+^) [53,54]. Accumulating evidence has indicated that activated Th1 and Th17 cells are capable of producing interleukin (IL)-17, IL-21, IL-22, granulocyte and macrophage colony stimulation factor (GM-CSF), interferon (IFN)-γ, and tumor necrosis factor (TNF)-α, are the main constituents of CD4^+^ T cells that drive MS development [55,56].

**Figure 2:**
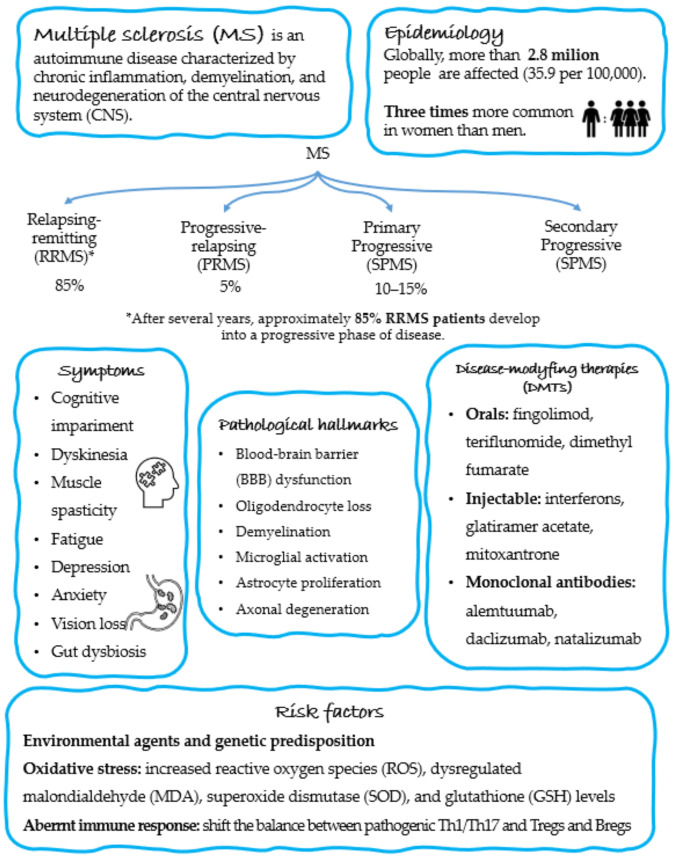
Characteristic and epidemiology of MS. It presents the division of MS into subtypes with their percentage occurrence, a list of the most common symptoms, current use of DMTs, identified pathological hallmarks, and general risk factors.

To better understand the immunopathology of MS, it is necessary to work with animal models of MS that mimic many aspects of the disease. The most commonly used model is experimental autoimmune encephalomyelitis (EAE), which is induced in rodents by active immunization with myelin peptides or via adoptive transfer of activated myelin-specific CD4^+^ T cells in naive recipients [57]. Based on EAE studies, it is assumed that Th1 cells producing IFN-γ were assumed to have a pathogenic role, while Th2 cells mainly producing IL-4 and IL-10 exert modulatory functions and have a protective role [58]. It was reported that mice lacking IL-23 production, the main cytokine engaged in Th17 differentiation, were protected from EAE development [59], while the transfer of myelin-specific Th17 cells into naive recipient mice induced neuroinflammation [60]. Furthermore, IL-17 knockout mice showed ameliorated symptoms of EAE [61]. In contrast to Th cells, Tregs are key players in immune regulation and inhibition of autoreactive immune cells. After activation, Tregs start their suppressive functions through the release of anti-inflammatory cytokines, such as IL-10 and transforming growth factor (TGF)-β [62]. Active transfer of Tregs alleviated the EAE symptoms, while genetic ablation led to the worsening course of the disease [63]. Both Th17 and Tregs were shown to frequently occur in the human intestine, while commensal microbiota can induce the differentiation of Th cells. Furthermore, it was recently shown that enhanced Th17 cell numbers in the intestine correspond with perturbations of the gut microbiota set and severe disease activity in people with MS. Summarize of the pathogenesis and epidemiology of MS are represented in Figure 2.

## 3. Gut Dysbiosis in MS

Changes in gut microbiota composition [64,65,66], bacteria-derived metabolites [67,68,69], intestinal permeability [70,71], and enteric nervous system functions [72] have been repeatedly described in MS patients. Studies on an animal model of MS have shown that the disruption of the intestinal barrier occurs soon after the induction of EAE [73,74].

Miyake et al. [66] analysis of the bacterial 16S rRNA gene by using a high-throughput culture-independent pyrosequencing method provided evidence of moderate dysbiosis in the structure of gut microbiota in Japanese RRMS patients. They found 21 species that showed significant differences in relative abundance between the RRMS and healthy subjects samples. They also reported differences in the bacterial composition between glatiramer acetate (GA)-treated and non-treated patients and revealed an increase in the *Akkermansia*, *Faecalibacterium*, and *Coprococcus* genera after vitamin D supplementation [66]. Jangi et al. [75] reported that in MS patients, there is an increase in *Methanobrevibacter* and *Akkermansia* and decreases in *Butyricimonas*, and correlate with variations in the expression of genes involved in the dendritic cell maturation, interferon signaling, and NF-kB signaling pathways in circulating T cells and monocytes. Patients on DMT show increased abundances of *Prevotella* and *Sutterella* and decreased *Sarcina* compared with untreated patients [75].

The vast majority of studies focus only on patients in the RR phase of MS. Currently, there is a lack of studies that would analyze changes in the gut microbiota in patients in the progressive phase. According to the available literature, a recently published study of dysbiosis in progressive MS patients in a Russian cohort reported that the relative domination of three bacterial phyla, *Akkermansia munciniphila*, *Gemminger*, and *Ruminococcacea* increased. Moreover, they suggest a common mechanism linking dysbiosis and disease pathogenesis, both in RRMS and SPMS patients [76].

Overall, patients with MS usually have gut dysbiosis and often reduced numbers of *Faecalibacterium* and *Prevotella* and increased amounts of *Akkermansia*. A summary of studies reporting dysbiosis in MS patients is presented in Table 1.

## 4. Effect of Probiotic Supplementation on EAE/MS

### 4.1. Animal Studies

Studies on EAE models have demonstrated that the commensal microbiota is an important agent in cognition improvement. The use of sophisticated strategies to manipulate the gut microbiome allowed scientists to verify the consequences of these changes on CNS and brain functions [85]. One of the simplest methods to analyze the influence of intestinal microorganisms on the CNS is to examine events when the microbiota is absent. The influence of gut bacteria on cognitive functions has been confirmed in research on germ-free animals. Germ-knock-out animals have improper brain development, manifested by altered neuronal plasticity [86], neurotransmission [87], limited myelination, and neurotrophin expression [88], as well as showed miscellaneous behavioral disorders [85]. Indeed, the absence of microbial factors in germ-knock-out or antibiotic-treated mice compared with wild-type animals (specific pathogen-free) resulted in diminished immune cell infiltration into CNS, leading in consequence to alleviate the overall course of EAE [89,90]. Furthermore, it is also reported probiotics may ameliorate the EAE course by decreasing IL-6 and preventing infiltration of Th17 cells into the CNS [91].

Depending on the phyla of a given probiotic strain, the influence on the autoimmunity of the EAE model can be different. Administration of *B. fragilis* [92], *P. histicola* [93], various species of *Lactobacillus* (*L. paracasei plus*, *L. plantarum* [94], *L. Reuteri* [95], and *L. helveticus* [91]), or multi-strain probiotics [96] has demonstrated to ameliorate the EAE symptoms and diminish the ongoing inflammation [97]. There are some probiotic bacteria, such as *L. casei* and *L. ruteri,* lead to increased production of pro-inflammatory cytokines, while other strains, such as *B. animalis*, *L. plantarum*, and *L. paracasei,* showed the opposite effect [98]. Hence, whether the gut microbiota will have a beneficial or negative effect on the course of EAE depends primarily on the type of bacterial strains and their amount. In one study, Yokote et al. [99] showed that variation in the intestinal microflora due to oral administration of antibiotics mix of kanamycin (1 mg/mL), colistin (2000 U/mL), and vancomycin (0.1 mg/mL), altered the composition of gut microflora, diminished the severity of disease symptoms and ameliorated the EAE development.

In turn, Kwon et al. [96] reported that oral administration of IRT5 probiotics ameliorated the progression of EAE by reducing MOG-reactive T cell proliferation and pro-inflammatory cytokine levels (IL-17, IFN-γ, and TNF-α), while enhancing IL10^+^ or/and FoxP3^+^ Tregs. Supplementation of IRT5 probiotics after EAE development significantly delayed disease onset, although it failed to completely suppress disease progression. These results suggest a potential application of IRT5 probiotics as a prophylactic modality or dietary supplements to modulate the progression of EAE and perhaps MS [96].

In general, the animal studies well elucidated that supplementation with specific probiotics may have an essential impact on the level of pro- and anti-inflammatory markers [96,100,101], ameliorate the EAE symptoms [102] and delay the onset of the disease [93,96,103]. Furthermore, probiotics effectively increase the level of Tregs (CD4^+^CD25^+^FoxP3^+^) and regulate the balance of Th1/Th17 and Th2 cytokines in EAE models [101,104]. The therapeutic role of probiotic administration in a multiplicity of EAE models has been described in Table 2.

### 4.2. Human Studies

The discovery that the gut microflora is able to induce pro- and anti-inflammatory effects has raised substantial interest in the supplementation of different bacteria strains for the treatment of CNS inflammation. To investigate the gut–CNS axis in MS, it is central to identify the mechanisms that link the gut microenvironment to CNS inflammation. Although there is still little known about the underlying mechanisms of gut-CNS interactions, the immunomodulatory properties of probiotics and their beneficial effects on diseases such as MS have been described in several studies. Currently, there are only a few clinical trials on the effects of probiotics on symptoms and MS development, as well the obtained data is limited to measuring the parameters of inflammation and oxidative stress.

One of the clinical trials showed that oral administration of probiotic VSL3 (a probiotic mixture containing 3 × 10^11^ CFU/g of viable lyophilized bacteria including three strains: *Lactobacillus*, *Bifidobacterium*, and *Streptococcus*, currently sold under the brand names Visbiome or Vivomixx) was associated with inducing an anti-inflammatory peripheral innate immune response in MS patients. Administration of VSL3 was associated with the diminished relative frequency of intermediate monocytes (CD14^high^/CD16^low^), as well as decreased mean fluorescence intensity (MFI) of human leukocyte antigen–antigen D related (HLA-DR) on myeloid-derived dendritic cells (CD45^+^/LIN^−^/CD11c^+^) in MS patients in comparison to the control group [106]. On the other hand, discontinuation of VSL3 induced a pro-inflammatory immune response characterized by increased frequency of inflammatory monocytes (CD14^low^/CD16^high^) and decreased frequency of IL-10+ Tregs, as well as the diminished relative frequency of CD39^+^/CD127^low^/CD25^high^Tregs in controls compared to MS subjects. However, the observed immunomodulatory effects did not persist after the discontinuation of VSL3 supplementation [106].

Probiotics administration may ameliorate disability and mental health symptoms by regulating anti-inflammatory response [107] and enhancing serotonin levels [108] in the brain. Study Kouchaki et al. [109] demonstrated that the use of probiotic capsules for 12 weeks among subjects with MS had favorable effects on the expanded disability status scale (EDSS) and parameters of mental health, including the Beck depression inventory (BDI), general health questionnaire-28 (GHQ-28), and the depression anxiety and stress scale (DASS). In another study, Mohammadi et al. [110] also observed an improvement in GHQ-28 and DASS after the 6-week administration of probiotics containing *L. acidophilus* LA5 and *B. lactis* BB12 strains (1 × 10^7^ CFU/g each). Similar results were obtained by Salami et al. [111], showing a significant influence of probiotic consumption on sanity health. Additionally, Rahimlou et al. [112] reported that supplementation with probiotics also reduces the fatigue severity scale (FSS) and pain rating index (PRI) in MS patients.

Besides the improvement in the parameters of mental health, probiotic supplementation also significantly affects the level of inflammatory and oxidative stress markers. It has also been shown that probiotic administration significantly diminished the high-sensitivity C-reactive protein (hs-CRP) and IL-6 levels, as well as an enhanced concentration of nitric oxide (NO) and IL-10 [111]. In another study, the supplementation of Lactobacillus strains prevented and delayed the clinical signs in the EAE model of MS. In this study, the probiotic supplement led to decreased levels of the pro-inflammatory cytokines (TNF-α, IFN-γ, and IL-17) and increased levels of the anti-inflammatory cytokine IL-10 [94]. Secher et al. [74] found that probiotic administration decreased IL-6 and improved clinical symptoms in EAE. Tankou et al. [78] reported that the administration of a probiotic supplement diminished frequencies of Th1 and Th17 in both the control group and MS patients.

Salami et al. [111] proved that taking the supplement led to a significant reduction in insulin, homeostasis model of assessment-estimated insulin resistance (HOMA-IR), MDA, and 8-hydroxy-2′-deoxyguanosine (8-OHdG) levels, and increased SOD, total antioxidant capacity (TAC) levels but did not affect GSH plasma level. In the next study, it was demonstrated that the administration of probiotics for 12 weeks led to decreased MDA and HOMA-IR and increased GSH levels [113].

What is more, there are also studies reporting that the administration of probiotics may influence the gene expression level. As an example, Tamtaji et al. showed that 12-week supplementation of probiotic capsules containing *L. acidophilus*, *L. casei*, *B. bifidum*, and *L. fermentum* (2 × 10^9^ CFU/g of each strain) down-regulated gene expression of IL-8 and TNF-α mRNA in peripheral blood mononuclear cells (PBMCs) of MS patients in comparison to the placebo group [113]. This study shows that proper supplementation with probiotics may regulate gene expression, which is a potentially powerful tool for influencing many molecular pathways that participate in the pathogenesis of MS. On the other hand, more research into the influence of probiotics on gene expression is needed, as well as detailed analysis confirming alterations at the protein level.

**Figure 3:**
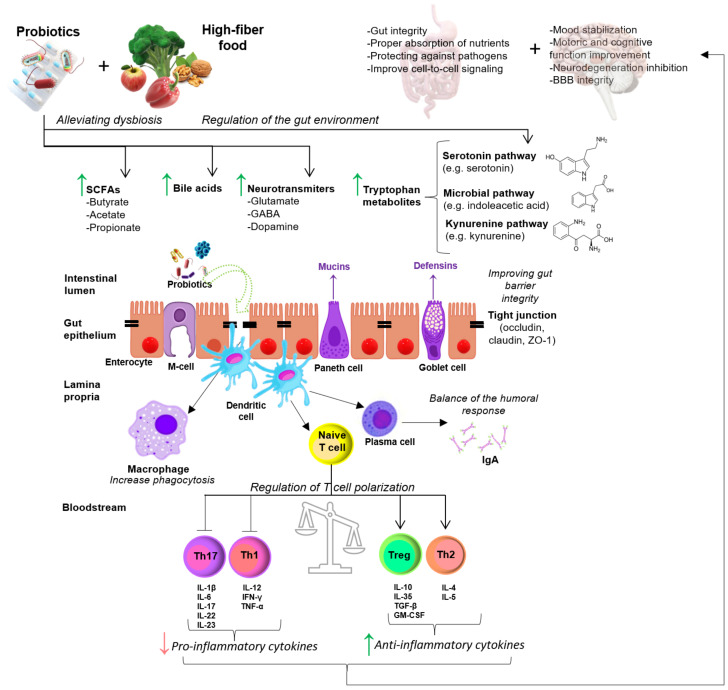
Potential favorable effects of probiotic supplementation on the molecular mechanisms of MS pathogenesis. Probiotics supplementation as well as high-fiber food intake, may affects the gut microflora and inhibit the increment of pathogens by inducing the host’s production of defensin and IgA by Goblet cells and plasma cells, respectively. Supplementation with appropriate amounts of probiotics causes an increase in intermediate metabolites, neurotransmitters (e.g., GABA, glutamate, and dopamine), SCFAs, and bile acids, which have a positive effect on the functioning of the intestines (e.g., proper absorption of nutrients) and the functioning of the brain (e.g., activation of serotonin and kynurenine pathways). Probiotics may be able to strengthen the gut barrier by maintaining tight junctions (e.g., occludin, zonulin, and claudin) and inducing mucin formation by Paneth cells. Probiotics via dendritic cell stimulation may regulate CD4^+^ T cell polarization and differentiation, leading to the production of pro-inflammatory (IL-1β, IL-6, IL-12, IL-17, IL-22, IL-23, and TNF-α) or anti-inflammatory (IL-4, IL-5, IL-10, IL-35, TGF-β, and GM-CSF) cytokines. Abbreviations: BBB—blood-brain barrier; GABA—gamma-aminobutyric acid; GM-CSF—Granulocyte-macrophage colony-stimulating factor; IFN-γ—interferon γ; IgA—immunoglobulin A; SCFAs—short-chain fatty acids; Th—helper T cells; IL—interleukin; TNF-α—tumor necrosis factor α; TGF-β—transforming growth factor β; ZO-1—zonula occludens-1.

Taken together, it seems, through modulation of gut microbiota, the probiotic treatment may improve clinical symptoms by a balance in inflammatory and anti-inflammatory responses in MS patients. Further, decreased oxidative stressors might be involved in controlling the clinical symptoms in patients with MS and EDSS parameters [114]. Overall, the administration of probiotic bacteria may influence motor and mental behaviors by modulation of inflammatory and oxidative biomarkers in patients with MS. Probiotic supplements could be a new strategy for improving and controlling MS severity. All described clinical trials on probiotics administration in MS patients are summarized in Table 3. The potential favorable effects of probiotic supplementation on the molecular mechanisms of MS pathogenesis are summarized in Figure 3.

## 5. Concluding Remarks and Future Perspectives

Promptly growing evidence confirms the GBA’s role in the pathogenesis of MS, with the intestine microbiome as a crucial player. Nevertheless, more research is needed to better clarify molecular pathways connecting gut and brain functions and how they impact CNS autoimmunity. Indeed, research on probiotics modulating the GBA pathways has been intensively investigated, however, it should be emphasized that the preclinical or clinical evidence on the beneficial effects of probiotics on CNS is still insufficient and arouses much controversy. Although the current evidence of the effects of probiotics on the CNS is based largely on animal studies and is limited to effects on cognition and immune cell infiltration, it provides an interesting source of data for further research on CNS inflammation and degeneration in MS patients.

Despite the promising immunomodulatory effects of these assisted therapies, only barely data exists on their impacts on MS development. Nonetheless, we still trust that in the nearby future, targeting the gut microbiota may be a favorable addition to approved DMTs. Currently, probiotic supplements are quite often introduced into treatment by patients themselves due to their readily available over-the-counter medications. While they do not pose a direct threat to life and do not cause serious side effects, the lack of adequate regulation in their controlled consumption only underlines the need for more clinical trials. Looking forward, gut microbiome-related therapeutics will presumably serve as an essential element for personalized medicine in the treatment of MS. This will require a comprehensive understanding of the exact role of the gut microbiome and the complex network of relationships between various bacterial strains and their role as a key element of the GBA pathway. This unconventional approach could also support monitoring responses to treatments and may seriously profit young people suffering from MS. Hence, a better understanding of the role of the GBA pathway and the impact of gut microbes on the CNS in MS patients may, in the future, result in a chance to develop new therapies using probiotic mixtures that will alleviate the course of the disease. Thus, there is a considerable need to conduct more research on the effects of probiotic administration on CNS, especially on MS patients.

## Figures and Tables

**Table 1:** Alterations in the gut microbiota of MS patients.

Subjects	Altered Genera in MS	Study
RRMS (*n* = 20) CTR (*n* = 40)	↑: *Streptococcus*, *Eggerthella* ↓: *Faecalibacterium*, *Prevotella*, *Anaerostipes*	Miyake et al., Japan (2015) [66]
RRMS (*n* = 60) CTR (*n* = 43)	↑: *Akkermansia*, *Methanobrevibacter* ↓: *Butyricimonas*, *Collinsella*, *Slackia*, *Prevotella*	Jangi et al., USA (2016) [75]
RRMS (*n* = 71) CTR (*n* = 71)	↑: *Akkermansia*, *Acinetobacter*, *Calcoaceticus* ↓: *Parabacteroides*	Cekanaviciute et al., USA (2017) [77]
RRMS (*n* = 9) CTR (*n* = 13)	↑: *Lactobacillus * ↓: *Akkermansia*, *Blautia*	Tankou et al., USA (2018) [78]
RRMS (*n* = 17) CTR (*n* = 17)	↑: atypical *E coli*, *Enterobacter* sp. ↓: *E. coli*	Abdurasulva et al., Russia (2018) [79]
RRMS (*n* = 19) CTR (*n* = 23)	↑: *Actinomyces*, *Eggerthella*, *Anaerofustis*, *Clostridia XIII*, *Clostridium III*, *Faecalicoccus*, *Streptococcus* ↓: *Butyricicoccus*, *Faecalibacterium*, *Dialister*, *Gemmiger*, *Lachnospiraceae*, *Subdolibacterium*	Forbes et al., Canada (2018) [80]
RRMS (*n* = 13) CTR (*n* = 14)	↑: None ↓: *Prevotella*	Oezguen et al., USA (2019) [81]
RRMS (*n* = 26) CTR (*n* = 39)	↑: *Bacteroidetes* ↓: *Coprococcus*, *Firmicutes*, *Paraprevotella*, *Ruminococcaceae*	Choileáin et al., USA (2020) [82]
RRMS (*n* = 26) SPMS (*n* = 12) CTR (*n* = 38)	↑: *Akkermansia* in SPMS, *Streptococcus* in RRMS, *Collinsella* in RRMS and SPMS ↓: *Coprococcus*, *Roseburia* in RRMS and SPMS, *Lachnospira* in RRMS	Saresella et al., Italy (2020) [69]
RRMS (*n* = 129) CTR (*n* = 58)	↑: *Lawsonella* ↓: *Faecalibacterium prausnitzii*, *Bacteroides fragiils*, *Eubacterium rectale*, *Butyrivibrio*, *Clostridium*, *Coprococcus*, *Roseburia*	Levi et al., Israel (2021) [83]
RRMS (*n* = 199) Progressive MS (*n* = 44) CTR (*n* = 40)	↑: *Clostridium*, *Bacteroides*, *Gemella*, *Akkermansia* in RRMS and progressive MS ↓: *Prevotella* and *Dorea* in RRMS and progressive MS	Cox et al., USA (2021) [84]

Abbreviations: CTR—control group; RRMS—relapsing-remitting multiple sclerosis; SPMS—secondary progressive multiple sclerosis.

**Table 2:** Effect of probiotic supplementation on different EAE animal models.

Model	Intervention	Duration	Measurements	Major Findings	Study
PLP-induced EAE in SJL/J female mice; MOG-induced EAE in C57BL/6 female mice (7 weeks old, *n* = 15 per group)	Administration groups (G): G1: Control (saline/peptone, orally) G2: *L. casei* strain Shirota (orally, once daily, 0.6–1.2 × 10^9^ CFU)	50 days	-Evaluation of neurological symptoms; -Histopathological changes in the spinal cord; -mRNA and protein level: IL-10, IL-17A, and IFN-γ; -Cytometric analysis of cell surface antigens: anti-CD3, anti-CD4, anti-CD8, and anti-CD25. Material: inguinal lymph nodes (ILN) and spleen.	-Improved neurological symptoms in the PLP model; -Slightly increased IL-10 level in ILN; -The enhanced percentage of CD4^+^/CD25^+^ (Tregs) in ILN and spleen; -Increased level of CD3^+^/CD8^+^ (Tcyt) in the spleen; -Elevated concentration of IL-17A and IL-10 in ILN.	Kobayashi et al., (2012) Japan [104]
MOG-induced EAE in C57BL/6 female mice (6–8 weeks old, *n* = 10 per group)	G1: Control (PBS, orally) G2: IRT5 probiotics powder: *L. casei*, *L. acidophilus*, *L. reuteni*, *B. bifidum*, and *S. thermophilus * (orally, once daily, 1 × 10^8^ CFU of each strain, final 5 × 10^8^ CFU)	30 days	-Clinical condition and symptoms using hematoxylin and eosin test staining; -mRNA level: IL-1β, IL-2, IL-4, IL-6, IL-10, IL-12, IL-17A, and TGF-β; -Cytometric analysis of cell surface antigens: anti-B220, anti-Gr1, anti-CD11b, anti-CD11c, and anti-CD4; intracellular cytokines: anti-IL-12, anti-IL-10, anti-IL-17, anti-IFN-γ, anti-Foxp3, and anti-TNF-α. Material: spinal cord.	-Inhibited development and progression of EAE; -Delayed onset of EAE; -Suppressed EAE incidence; -Decreased the clinical symptoms of EAE; -Reduced lymphocyte infiltration in the spinal cord; -Decreased levels of Gr1^+^ or/and CD11b^+^ monocyte and CD4^+^ T cells in the spinal cord; -Suppressed expression levels of pathogenic cytokines: IL-1β, IL-2, IFN-γ, TNF-α, and IL-17; -Enhanced production of IL-10 in CD4^+^ T cells and CD11c^+^ dendritic cells; -Slightly increased level of B220^+^ B cells; -Mitigated Th1/Th17 polarization while inducing IL-10^+^ producing CD4^+^ T cells in draining lymph nodes; -Down-regulated expression levels of IL-6, IFN-γ and TNF-α at mRNA level by CD4^+^ T cells; -Enhanced generation of CD4^+^/FoxP3^+^ Tregs at the site of inflammation.	Kwon et al., (2013) Republic of Korea [96]
EGM-induced EAE in male Wistar rats (3 months old, total *n* = 122 per 4 groups)	G1: Control (saline, subcutaneously) G2: Control (saline, intragastric) G3: Glatiramer acetate (GA) (subcutaneously, 4 mg/kg/day) G4: *E. faecium* L3 (intragastrically, 8 CFU/mL)	28 days	-Blood cell phenotyping by flow cytometry: anti-CD3, anti-CD4, anti-CD8, anti-CD16, anti-CD25, anti-FoxP3, anti-CD45RA; -Evaluation of neurological symptoms. Material: spinal cord and whole blood	-Decreased severity and disease duration of EAE animals; -Reduced number of (CD4^+^/CD25^+^/FoxP3^+^) Tregs and NK cells.	Abdurasulova et al., (2016) Russia [103]
MOG-induced EAE in C57BL/6 female mice (8–10 weeks old, *n* = 8 per group)	G1: Control (saline, orally) G2: *L. plantarum* (intragastric, once daily, 1 × 10^9^ CFU) G3: *B. animalis* (intragastric, once daily, 1 × 10^9^ CFU) G4: both probiotics	22 days	-Clinical score evaluation; -Body weight control; -Histopathology of the spinal cord; -Evaluate the proliferative activity of isolated splenic T cells using a Brdu assay; -Determination of Tregs by flow cytometry using anti-CD4, anti-CD25, and anti-FoxP3; -Protein level: IL-4, IL-6, IL-10, IL-17, IFN-γ, TGF-β; -mRNA level: FoxP3, T-bet, GATA3, and RORγt. Material: spinal cord, spleen, brain, and peripheral lymph nodes.	-Induced polarization of CD4^+^ T cells toward anti-inflammatory Tregs (CD4^+^/CD25^+^/Foxp3^+^); -Suppressed autoreactive T cells proliferation; -Inhibited leukocyte infiltration into CNS; -Ameliorated EAE condition by favoring Th2 and Treg differentiation; -Inhibited differentiation of Th1 and Th17 cells; -Increased level of IL-6, IL-17, IFN-γ, and diminished concentration of IL-4, IL10 and TGF-β in splenocytes and lymph nodes.	Salehipour et al., (2017) Iran [101]
MOG-induced EAE in C57BL/6 female mice (8–12 weeks old, *n* = 30–40 per group)	G1: Control (PBS, orally) G2: *E.coli* Nissle 1917 (ECN) (orally, one daily, 1 × 10^8^ CFU) G3: archetypal *E.coli* strain MG1655 (orally, one daily, 1 × 10^8^ CFU)	30 days	-In vivo and ex vivo intestinal permeability assessment; -mRNA level: ZO-1, claudin-8, IL-6, Reg3β, and Reg3γ; -Protein level: IFN-β, IL-17, GM-CSF. Material: serum, ileum, colon, brain, spinal cord, and lymph nodes.	-ECN reduced the severity of EAE; -ECN treatment protects from EAE-mediated alteration of the intestinal barrier function; -Reduced migration of CD4^+^ T cells from the periphery to the CNS during the acute phase; -Increased production of IL-10 by MOG-specific CD4^+^ T cells.	Secher et al., (2017) France [74]
PLP-induced EAE in HLA-DR3.DQ8 double transgenic and C57BL/6, both male and female mice (8–12 weeks old, *n* = 4–8 mice per group)	G1: Control (PBS, orally) G2: TSB media (orally) G3: *P. histicola* (orally, one daily, 10^8^ CFU) G4: Copaxone^®^ (GA) (subcutaneously, 100 μg every day) G5: Copaxone^®^+ *P. histicola*	14 days	-Evaluation of clinical EAE scores; -Clinical condition and symptoms using hematoxylin and eosin test staining; -Evaluation of gut microbiota composition; -Cytometric analysis of cell surface antigens: anti-CD4 and anti-CD25, intracellular expression of FoxP3+ and IL-10; Material: fecal pellets, brain, and spinal cord.	-Significantly reduced severity score and delayed onset of disease; -Increased number of CD4^+^/FoxP3^+^ Tregs in periphery and gut; -Reduced frequency of IFN-y and IL-17-producing CD4^+^ T cells in the CNS; -*P. histicola,* together with Copaxone^®^, more effectively suppressed disease compared to either treatment alone.	Shahi et al., (2019) USA [93]
SCH-induced EAE in female Dark Agouti (DA) rats (8–10 weeks old, *n* = 5 per group)	G1:Control (MRS Broth, orally, medium for *Lactobacillus* spp.) G2: *L. brevis* BGZLS10-17 (high GABA-producing strain) (subcutaneously, one daily, 1 × 10^8^ CFU)	30 days	-Neurological symptoms assessment. Material: spinal cord.	-Ameliorated severity score of EAE model (G2) after *L. brevis* intake.	Sokovic Bajic et al., (2019) Serbia [102]
MBP-induced EAE in female SJL/J mice (6–9 weeks old, *n* = 3 per group)	G1: Control (medium, orally) G2: *S. thermophilus* 285 (orally, one daily, 1 × 10^8^ CFU)	14 days	-Cytokine level analysis: IL-1β, IL-2, IL-4, IL-5, IL-6, IL-10, GM-CSF, TNF-α, and IFN-γ using Bioplex system. Material: spleen.	-Increased level of IL-4, IL-5 and IL-10 cytokines and diminished levels of IL-1β and IFN-y.	Dargahi et al., (2020) Australia [100]
TMEV-infected susceptible female SJL/J mice (6–8 weeks old, *n* = 5–10 per group)	G1: Sham mice G2: Sham mice + Vivomixx (orally, 3 × 10^8^ CFU) G3: TMEV-mice G4: TMEV-mice + Vivomixx Vivomixx (L. paracasei, *L. plantarum*, *L. acidophilus*, *L. delbruckeii* subspecies *bulgaricus*, *B. longum*, *B. infantis*, *B. breve*, and *S. thermophilus*).	15 days	-Assessment of the motor functions; -Measurement of bacteria-derived SCFAs; -mRNA level: IL-1β, IL-6, TNF-α, IL-4 and IL-10 in spinal cord; -Estimation of the level of Tregs and Bregs population; -Microglial morphology; -Cytometric analysis of cell surface antigens: anti-CD4, anti-CD8, and anti-CD39; -Identification of the gut microbiota community changes. Material: plasma, brain, spinal cord, spleen, and mesenteric lymph nodes.	-The increased abundance of *Bcteroidetes*, *Actinobacteria*, and *Tenericutes*; -Improved motor disability; -Reduced microgliosis, astrogliosis, and leukocyte infiltration; -The enhanced presence of Bregs (CD19^+^/CD5^+^/CD1d^high^) in the CNS; -Diminished IL-1b and IL-6 gene expression in spinal cord; -Promoted IL-10 gene expression; -Increased plasma level of butyrate and acetate levels; -Restricted IL-17 production by Th17-polarized CD4^+^ T cells from mesenteric lymph nodes.	Mestre et al., (2020) Spain [97]
Cuprizone-induced mouse model of demyelination in C57BL/6 female mice (8–10 weeks old)	G1: Control G2: Cuprizone control G3: Probiotic control G4: *L. casei* (oral administration, 1 × 10^9^ CFU) for 4 weeks, then cuprizone for 4 weeks G5: Cuprizone for 4 weeks, then *L. casei* for 4 weeks G6: Cuprizone for 4 weeks, then *L. casei* for 4 weeks with vitamin D3 (20 IU per day)	28 days	-Assessment of the motor behaviors; -Y-maze test for spatial memory and learning; -mRNA expression: IDO-1, miR-155, and miR-25; -Protein level: IL-17 and TGF-β. Material: brain, blood.	-*L. casei* ameliorated the CPZ-induced motor impairment; -Decreased the mRNA expression of IFN-γ, IDO-1, and miR-155; -Increased serum level of TGF-β and miR-25; -*L. casei* can shift responses from Th17 to Tregs; -Reduced pro-inflammatory cytokines; -Diminished demyelinating symptoms.	Gharehkhani Digehsara et al., (2020) Iran [105]

Abbreviations: CD—cluster of differentiation; CFU—colony forming unit; CNS—central nervous system; CPZ—cuprizone model; EAE—experimental autoimmune encephalomyelitis; ECN—E. coli strain Nissle; EGM—glycol monomethyl ether; FoxP3—forkhead box P3; GA—glatiramer acetate; GATA3—GATA binding protein 3; GM-CSF—Granulocyte-macrophage colony-stimulating factor; IDO-1—indoleamine 2, 3-dioxygenase 1; IFN-γ—interferon γ; IL—interleukin; ILN—inguinal lymph nodes; MBP—myelin basic protein; MOG—myelin oligodendrocyte glycoprotein; NK—natural killer cells; PLP—proteolipid protein; Reg3β—regenerating islet-derived protein 3β; Reg3γ—regenerating islet-derived protein 3γ; RORγt—RAR-related orphan receptor γ; SCH—spinal cord homogenate; T-bet—T-box expressed in T cells; Tcyt—cytotoxic T cells; TGF-β—transforming growth factor β; Th—helper T cells; TMEV—Theiler’s murine encephalomyelitis virus; TNF-α—tumor necrosis factor α; Tregs—regulatory T cells; ZO-1—zonula occludens-1.

**Table 3:** Characteristic of the clinical studies concerning probiotic supplementation.

Subjects	Sex Ratio (M/F)	BMI (kg × m^−2^)	Average Age ± SD	Probiotic Bacteria	Dosage (CFU g^−1^)	Administration	Major Findings	Limitations	Study
RRMS (*n* = 40) (EDSS ≤ 4.5) Including: Placebo group (*n* = 20) and probiotic group (*n* = 20)	No data.	Placebo group: 24.7 ± 3.7 Probiotic group: 25.6 ± 4.6	Placebo group: 34.9 ± 8.9 Probiotic group: 32.8 ± 9.2	*L. acidophilus*, *L. casei*, *B. bifidum*, and *L. fermentum* Placebo group: starch	2 × 10^9^	Orally, once a day for 3 months	-Down-regulated gene expression of IL-8 and TNF-α in PBMCs compared with the placebo group.	-Lack of information about microbiota changes; -Small sample size; -No confirmation of changes in the proteins level of studied molecules (only gene expression results); -Lack of diet control.	Double-blind RCT Tamtaji et al., (2017) Iran [113]
RRMS (*n* = 60) (EDSS ≤ 4.5) Including: Placebo group (*n* = 30) and probiotic group (*n* = 30)	Placebo group: 5/25 Probiotic group: 5/25	Placebo group: 24.7 ± 3.3 Probiotic group: 25.4 ± 4.0	Placebo group: 33.8 ± 8.9 Probiotic group: 34.4 ± 9.2	*L. acidophilus*, *L. casei*, *B. bifidum*, and *L. fermentum* Placebo group: starch	2 × 10^9^	Orally, once a day for 3 months	-Improved EDSS, BDI, GHQ-28, and DASS scales; -Decreased serum insulin level; -Increased quantitative insulin sensitivity check index and HDL-cholesterol levels; -Diminished levels of hs CRP, plasma NO metabolites, and MDA.		Double-blind RCT Kouchaki et al., (2017) Iran [109]
Control group (CTR) (*n* = 13) RRMS on GA (*n* = 7) or untreated (*n* = 2)	No data.	CTR: 25.8 ± 4.1 MS: 31.1 ± 5.6	CTR: 35 ± 14 MS: 50 ± 10	VSL3 probiotics powder consisting of *Lactobacillus* (*L. paracasei*, *L. plantarum*, *L. acidophilus*, and *L. delbruckeii* subspecies *bulgaricus*), Bifidobacterium (*B. longum*, *B. infantis*, and *B. breve*) and *S. thermophilus* Brand name: Visbiome (USA) or Vivomixx (Europe).	3 × 10^11^	Orally, twice daily for 2 months.	-Diminished level of CD14^+^CD16^+^ and enhanced frequency of CD8^+^ T cells in MS patients; -Decreased MFI of HLA-DR on CD45^+^/LIN^−^/CD11c^+^ in MS patients; -The relative level of Th1 and Th17 cells were trending down in both controls and MS patients.	-Very small study and control group; -RRMS subjects (n = 2) were treated with glatiramer acetate during supplementation; -The subjects enrolled in this study were not on a dietary restriction; -No information about the gender of the subjects.	Clinical Trial Tankou et al., (2018) USA [106]
RRMS (*n* = 48) (EDSS ≤ 4.5) Including: Placebo group (*n* = 24) and probiotic group (*n* = 24)	Placebo group: 8/18 Probiotic group: 6/18	Placebo group: 24.5 ± 0.63 Probiotic group: 24.7 ± 0.55	Placebo group: 36.5 ± 1.44 Probiotic group: 34.8 ± 1.06	*B. infantis*, *B. lactis*, *L. reuteri*, *L. casei*, *L. plantarum* and *L. fermentum* Placebo group: maltodextrin	2 × 10^9^	Orally, once daily for 4 months.	-Markedly improves mental health parameters: BDI, GHQ-28, and DASS; -Reduced levels of hs-CRP, NO, and MDA; -Improved insulin resistance and lipid metabolism. -Decreased EDSS parameter.	-There is no information about the potential changes in bacterial strains.	Double-blind RCT Salami et al., (2019) Iran [111]
RRMS (*n* = 70) (EDSS ≤ 4.5) Including: Placebo group (*n* = 35) and probiotic group (*n* = 35)	Placebo group: 12/21 Probiotic group: 6/26	Placebo group: 24.55 ± 3.51 Probiotic group: 25.48 ± 4.54	Placebo group: 39.9 ± 8.76 Probiotic group: 42.15 ± 11.98	Protein probiotics powder consisting of the following: *B. subtilis*, *B. bifidum*, *B. breve*, *B. infantis*, *B. longum*, *L. acidophilus*, *L. bulgaricus*, *L. casei*, *L. plantarum*, *L. rhamnosus*, *L. helveticus*, *L. salivarius*, *L. lactis*, and *0S. thermophilus*. Placebo group: maltodextrin	2 × 10^9^	Orally, twice daily for 6 months.	-Greater improvement in mental health parameters: GHQ-28, BDI, FSS, PRI.		Double-blind RCT Rahimlou et al., (2020) Iran [112]

Abbreviations: BDI—Beck’s depression inventory; CD—cluster of differentiation; CFU—colony forming unit; DASS—depression anxiety stress scales; EDSS—expanded disability status scale; F—female; FSS—fatigue severity scale; GHQ-28—general health questionnaire 28; HDL—high-density lipoprotein; HLA-DR—human leukocyte antigen—DR isotype; Hs-CRP—high sensitivity C-reactive protein; IL—interleukin; M—male; MDA—malondialdehyde; MFI—mean fluorescence intensity; MS—multiple sclerosis; NO—nitric oxide; PBMCs—peripheral blood mononuclear cells; PRI—pain rating index; RCT—randomized clinical trial; RRMS—relapsing-remitting multiple sclerosis; Th—helper T cells; TNF-α—tumor necrosis factor α.
